# Meta-Analysis of Brand-Mixed Versus Brand-Unmixed Primary Total Hip Arthroplasty

**DOI:** 10.3390/jcm15207904

**Published:** 2026-10-13

**Authors:** Daniel Elias, Bryan J. M. van de Wall, Jörg Bucher, Björn-Christian Link, Pascal C. Haefeli

**Affiliations:** 1 Department of Orthopedic and Trauma Surgery, Lucerne Cantonal Hospital, 6004 Lucerne, Switzerland; bryan.vandewall@luks.ch (B.J.M.v.d.W.); joerg.bucher@luks.ch (J.B.); bjoern-christian.link@luks.ch (B.-C.L.); pascal.haefeli@luks.ch (P.C.H.); 2 Department of Health Science and Medicine, University of Lucerne, 6005 Lucerne, Switzerland

**Keywords:** total hip arthroplasty, brand-mixed components, implant compatibility, revision rates, meta-analysis

## Abstract

**Background/Objectives**: Mixing implant components from different manufacturers in primary total hip arthroplasty (THA) remains controversial because of concerns regarding implant compatibility and revision risk. This systematic review and meta-analysis aimed to compare revision outcomes between brand-mixed and brand-unmixed primary THA. **Methods**: A PRISMA-compliant systematic review of PubMed/MEDLINE and Embase identified studies comparing brand-mixed and brand-unmixed primary THA. Reported time-to-event estimates were pooled as hazard ratios (HRs), and revision-event counts as odds ratios (ORs), using random-effects models. **Results**: Six retrospective observational studies comprising 1,185,948 primary THAs were included. The time-to-event analysis did not identify a statistically significant difference in all-cause revision between brand-mixed and brand-unmixed THA (HR 1.03; 95% CI 0.97–1.08; I^2^ = 0%). The complementary crude revision-event analysis likewise did not identify a statistically significant difference (OR 1.01; 95% CI 0.83–1.24), although substantial heterogeneity was present (I^2^ = 93%). A post hoc exploratory sensitivity analysis excluding metal-on-metal and ceramic-on-metal bearings and brand-mixed stem–head constructs was based on two studies and yielded an OR of 0.97 (95% CI 0.79–1.20; I^2^ = 95%). Certainty of evidence was low for the time-to-event analysis and very low for the event-based analyses. **Conclusions**: Available observational evidence did not identify a statistically significant association between brand mixing and all-cause revision following primary THA. However, these findings do not establish equivalence, universal component compatibility, or the safety of specific brand-mixed combinations and should be interpreted in light of the observational study designs, residual confounding, and heterogeneity in implant constructs.

## 1. Introduction

The use of implant components from different manufacturers in primary total hip arthroplasty (THA)—commonly referred to as “mix-and-match” and hereafter termed “brand-mixed”—remains a subject of ongoing debate within the orthopedic community. In revision THA, brand-mixed constructs are often unavoidable due to the unavailability of original components or the need for case-specific implants not supplied by a single manufacturer. Retaining well-fixed components without signs of wear can reduce morbidity; for example, revising only the femoral stem while preserving a stable acetabular cup may substantially decrease operative burden compared with full component revision. Observational evidence from revision THA has not consistently demonstrated higher failure rates with brand-mixed constructs, which has contributed to consideration of this practice in primary THA [1,2,3].

The potential advantages of brand-mixed primary THA are compelling: surgeons may combine the best-performing components from different manufacturers to create constructs tailored to the patient’s needs. Although many implants share similar design principles (e.g., Müller-type straight stems), they often differ in critical details that may influence clinical outcomes. While outcomes of primary THA are generally excellent, these subtle variations may further improve results, particularly in patients with specific anatomical or functional requirements. For example, collared stems may reduce the risk of periprosthetic fracture [4,5,6,7], and the dimensional characteristics of monobloc cups can allow the use of larger femoral heads without increasing outer cup diameter or reducing polyethylene thickness, potentially enhancing range of motion and reducing dislocation risk while preserving acetabular bone [8]. Similarly, cups without metal backs may decrease the risk of psoas tendinitis due to the absence of sharp metal edges [9], and improved instrumentation with certain implants may limit soft tissue trauma. Beyond clinical outcomes, brand-mixed constructs may offer practical advantages: faster implantation of monobloc cups can improve surgical efficiency and potentially reduce costs, while engagement with multiple manufacturers may strengthen institutional resilience against supply chain disruptions and improve leverage in price negotiations.

**Figure 1:**
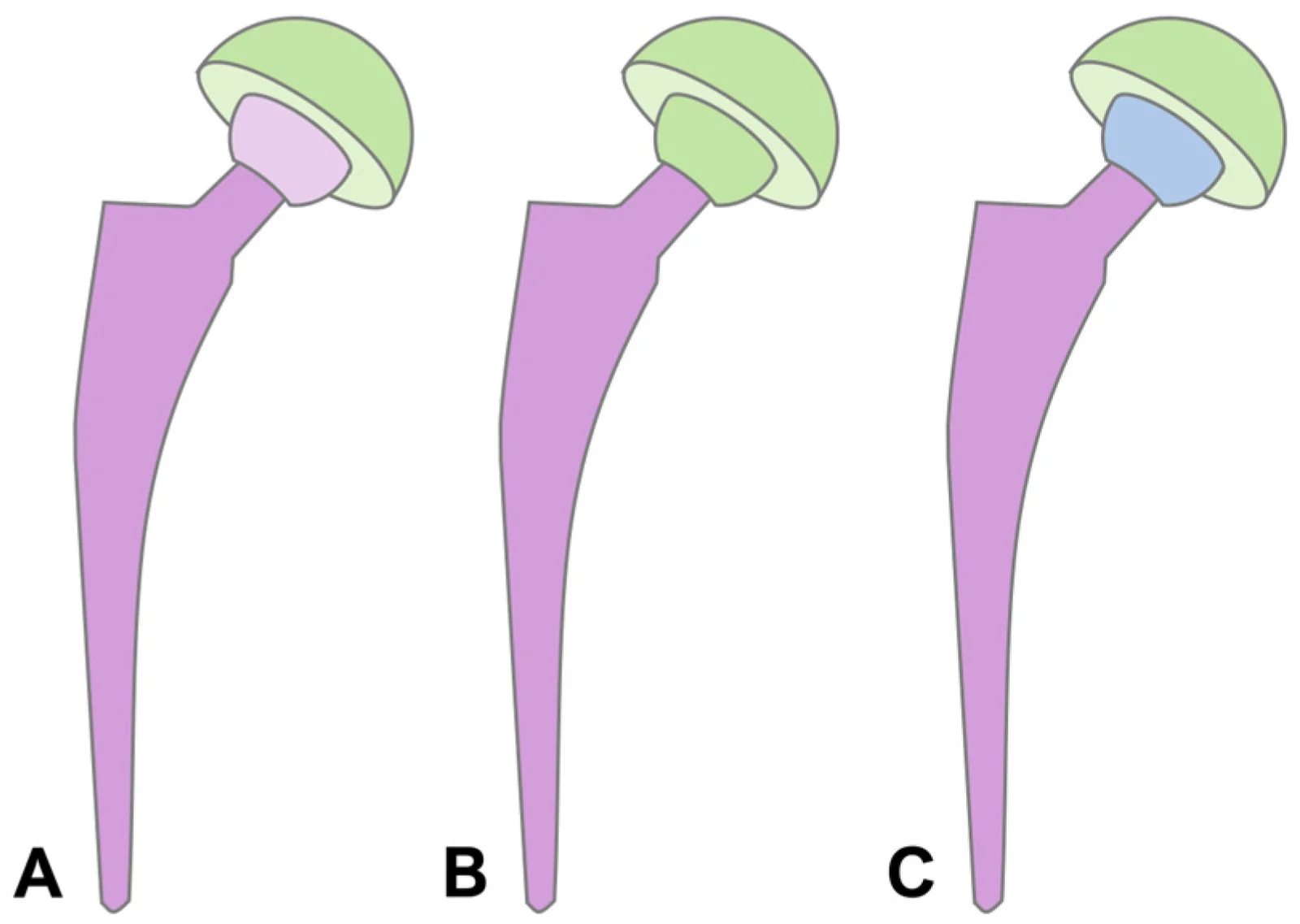
Brand-mixed combinations in primary total hip arthroplasty. (**A**) Head–cup combination. (**B**) Head–stem combination. (**C**) Stem–head–cup combination.

The most frequent brand-mixed combinations involve head–cup pairings (Figure 1A). Head–stem combinations (Figure 1B), once more common, have declined in popularity due to concerns regarding taper connection integrity, which may lead to fretting, wear, or ceramic head fracture. Although these risks are supported by biomechanical testing [10,11], clinical evidence does not consistently demonstrate inferior outcomes [1]. Brand-mixed stem–head–cup constructs, involving components from three different manufacturers (Figure 1C), are rare.

Criticism of brand-mixed primary THA focuses on the absence of standardized testing, regulatory approval, and long-term outcome data. Despite being considered off-label, brand-mixed constructs are widely used, with national joint registry data reporting prevalence rates ranging from 5% to 30% [12,13,14,15,16,17].

The primary aim of this systematic review and meta-analysis was to compare all-cause revision risk between brand-mixed and brand-unmixed implants in primary THA. Secondary aims were to evaluate reported indications for revision and functional outcomes. By quantitatively synthesizing the available revision data and descriptively evaluating additional outcomes, this study sought to better characterize the available clinical evidence regarding brand mixing in primary THA.

## 2. Materials and Methods

This systematic review and meta-analysis was conducted in accordance with the Preferred Reporting Items for Systematic Reviews and Meta-Analyses (PRISMA) 2020 statement (Supplementary PRISMA 2020 CHECKLIST) [18] and the Meta-analysis of Observational Studies in Epidemiology (MOOSE) guidelines [19]. Ethical approval was not required as no primary data were collected. The study protocol was registered with the International Prospective Register of Systematic Reviews (PROSPERO; CRD420251229930). Deviations from the registered analysis plan are described below.

### 2.1. Search Strategy and Selection Criteria

A systematic search was performed in PubMed/MEDLINE on 4 September 2024 and in Embase on 20 September 2024 to identify studies comparing primary THA constructs comprising components from different manufacturers with those comprising components from a single manufacturer. The searches were updated in PubMed/MEDLINE on 10 September 2026 and in Embase on 11 September 2026 using the same search strategy to identify subsequently published eligible studies. The literature uses heterogeneous terminology for the combination of components from different manufacturers, including “mix-and-match,” “mixed,” and “non-mixed” constructs. Therefore, the search strategy incorporated broader terms related to component mixing rather than relying on a single designation. For clarity and consistency, we use the terms “brand-mixed” and “brand-unmixed” throughout this review to distinguish constructs comprising components from different manufacturers from those comprising components from a single manufacturer, respectively. The complete search strategy is provided in the Supplementary Material (Table S1).

Two reviewers (DE/BJMvdW) independently screened titles and abstracts for eligibility. Studies were included if they met all of the following criteria:

1. Compared brand-mixed versus brand-unmixed components in primary THA;
2. Were randomized controlled trials or prospective/retrospective observational studies;
3. Reported all-cause revision as an outcome;
4. Included participants aged 18 years or older; and
5. Were published in English.

Studies were excluded if they focused on revision THA, had a follow-up of less than one year, were biomechanical or laboratory-based, lacked full-text availability, or were non-primary research articles (e.g., letters, commentaries, editorials, or case reports).

Full-text articles were then assessed by the same reviewers, with disagreements resolved through discussion or consultation with a third reviewer (PCH). Reference lists of included studies were screened to identify additional studies. Grey literature, conference abstracts, and trial registries were not systematically searched.

### 2.2. Data Extraction

Data extraction was performed independently by two reviewers (DE/BJMvdW) using a standardized data collection form. Additional information was requested from study authors when necessary. The specific component interfaces used to classify constructs as brand-mixed were extracted according to the definitions reported by the individual studies. Where both unmatched and propensity score–matched cohort data were reported, data from the propensity score–matched analysis were preferentially extracted to reduce the influence of measured baseline confounding. Where adjusted effect estimates were reported, the adjustment method and covariates included in the original analysis were also extracted.

The following study and baseline characteristics were extracted: first author, year of publication, country, study design, study period, length of follow-up, number of patients in each group, mean age, sex distribution, ASA score, body mass index (BMI), use of cementation, type of bearing surface, femoral head size, and history of previous operations. These characteristics are summarized in Table 1a–c.

Age data were extracted as reported by the individual studies, including mean age and the proportion of participants below study-specific age thresholds.

Outcome data included the number of revisions, reported hazard ratios (HRs) and corresponding 95% confidence intervals for all-cause revision, reasons for revision, functional outcomes, and operative duration.

All data were managed using Microsoft Excel (Microsoft Corporation, Redmond, WA, USA).

### 2.3. Study Outcomes

The primary outcome was all-cause revision following brand-mixed versus brand-unmixed primary THA.

Secondary outcomes included reported reasons for revision and their distribution between groups, as well as functional outcomes.

### 2.4. Quality Assessment

Methodological quality was assessed independently by two reviewers (DE/BJMvdW) using the Methodological Index for Non-Randomized Studies (MINORS) [20], as prespecified in the registered review protocol. Discrepancies were resolved by consensus. Further details are provided in the Supplementary Material (Table S2).

### 2.5. Certainty of Evidence Assessment

The certainty of evidence for the primary outcome was evaluated using the GRADE approach [21] across the five domains: risk of bias, inconsistency, indirectness, imprecision, and publication bias. Certainty was assessed separately for the time-to-event analysis, the crude revision-event analysis, and the post hoc exploratory contemporary-construct sensitivity analysis. As all included studies were observational cohort studies, the certainty of evidence started at “low” and could be downgraded further based on concerns within the GRADE domains. The certainty rating (high, moderate, low, or very low) was determined independently by two reviewers (DE/BJMvdW), with consensus reached through discussion.

A Summary of Findings table presenting the GRADE certainty ratings, pooled effect estimates, and reasons for downgrading is provided in the Supplementary Material (Table S3).

### 2.6. Statistical Analysis

Baseline characteristics were summarized descriptively as reported by the individual studies, without quantitative pooling or imputation of missing data.

The registered analysis plan specified pooling of revision-event counts as odds ratios. Because revision is a time-to-event outcome and crude event proportions do not account for differences in follow-up duration or censoring, a time-to-event meta-analysis using reported hazard ratios (HRs) was subsequently added and given greater interpretative emphasis. For studies reporting suitable HRs for all-cause revision, HRs were pooled using the generic inverse-variance method under a random-effects model and reported with 95% confidence intervals (CIs). Adjusted HRs were preferentially used where available; however, adjustment strategies differed among studies, and not all included HRs were multivariable-adjusted. Effect estimates were harmonized to represent brand-mixed versus brand-unmixed THA; HRs reported in the opposite direction were reciprocally transformed.

As the time-to-event analysis was not prespecified, the hierarchy for selecting among multiple eligible HR estimates was also determined post hoc. Where multiple HRs were available within a study, estimates considered most representative of contemporary brand-mixed constructs were selected for the principal analysis, preferentially avoiding potentially confounding construct characteristics where possible. Sensitivity analyses using alternative eligible HR estimates were performed to evaluate the robustness of the pooled result to effect-estimate selection.

The prespecified revision-event analysis was retained as a complementary analysis. For studies reporting extractable revision-event counts, all-cause revision was analyzed as a dichotomous outcome using the number of revisions and total number of THAs in each group. Effect estimates were pooled using the Mantel–Haenszel method under a random-effects model and reported as odds ratios (ORs) with 95% CIs. These estimates were based on aggregate event counts and therefore did not incorporate study-level multivariable adjustment; for Al-Jabri et al. [15], event counts from the propensity score–matched cohorts were used. Given the substantial heterogeneity observed in this analysis, a post hoc leave-one-out sensitivity analysis was performed by sequentially excluding each study to assess the influence of individual studies on the pooled estimate and heterogeneity.

A post hoc exploratory contemporary-construct sensitivity analysis of revision-event data was additionally performed, excluding metal-on-metal and ceramic-on-metal bearings and brand-mixed stem–head constructs. This analysis was intended to explore revision outcomes in implant configurations more representative of contemporary clinical practice.

Statistical heterogeneity was assessed using the I^2^ statistic and visual inspection of forest plots. Given the anticipated clinical and methodological heterogeneity among the included observational studies, random-effects models were used for all meta-analyses. A *p*-value < 0.05 was considered statistically significant. Formal assessment of publication bias using funnel plots or statistical tests was not performed because only six studies were included, limiting the reliability of such methods; publication bias was instead considered qualitatively as part of the GRADE assessment [21]. Potential sources of heterogeneity were additionally explored qualitatively based on differences in study populations, follow-up duration, implant characteristics, and analytical approaches. All statistical analyses were performed using Review Manager (RevMan), version 5.4.1 (The Cochrane Collaboration, Copenhagen, Denmark).

## 3. Results

### 3.1. Search Results

**Figure 2:**
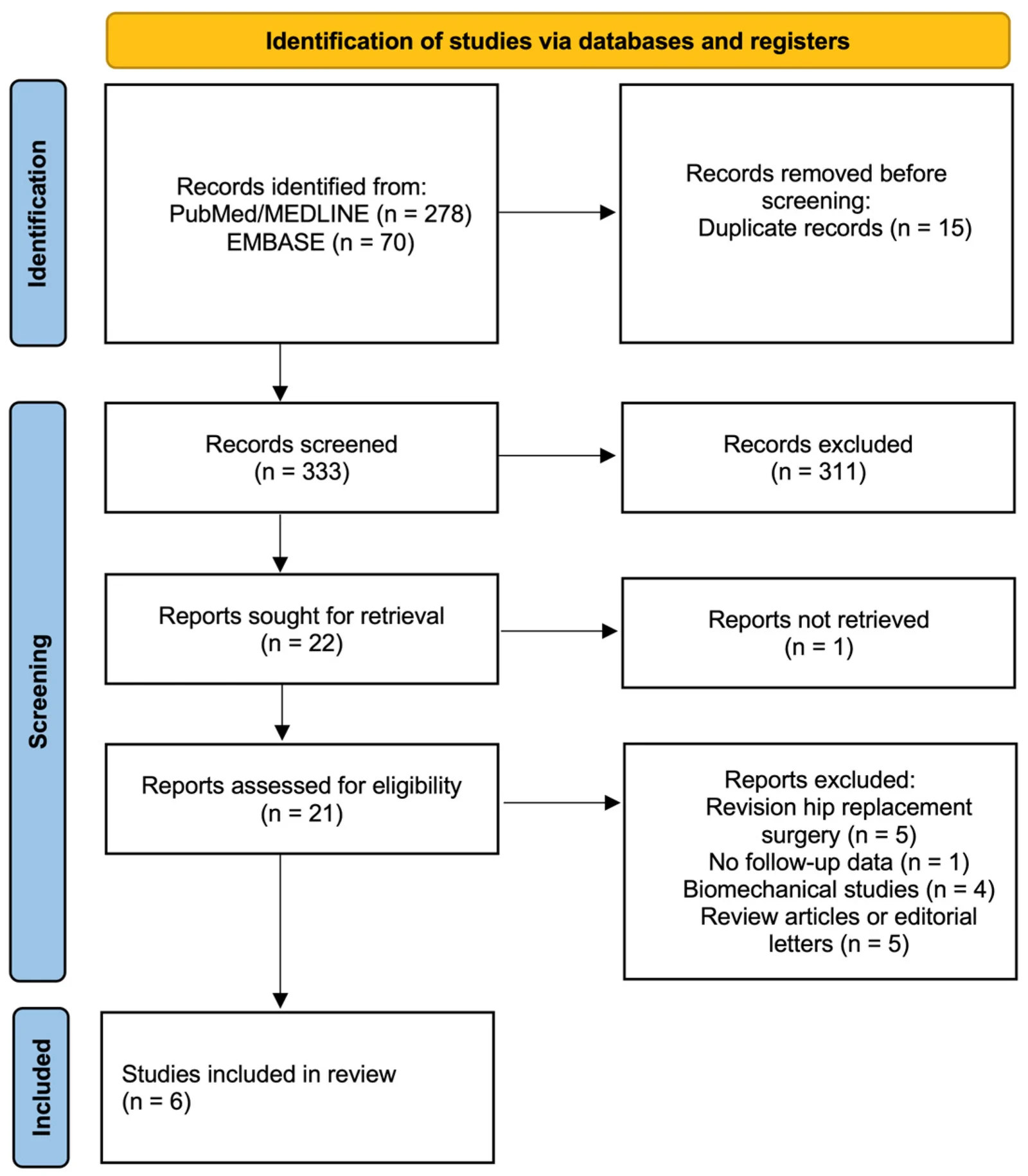
PRISMA flow diagram. PRISMA 2020 flow diagram illustrating the identification, screening, eligibility assessment, and inclusion of studies in the systematic review.

As shown in the PRISMA 2020 flow diagram (Figure 2), six retrospective cohort studies met the inclusion criteria. Four studies were identified in the original literature search, and two additional studies were identified through the updated searches conducted in September 2026. No randomized controlled trials or prospective observational studies meeting the eligibility criteria were identified. One potentially relevant study by Malcolm et al. [17] was excluded because it reported only prevalence and perioperative outcomes without longitudinal follow-up data on revision.

### 3.2. Study Characteristics and Baseline Demographics

Across the six included studies, 1,185,948 primary THA procedures were included in the extracted study populations, of which 151,253 (12.8%) were brand-mixed and 1,034,695 (87.2%) were brand-unmixed. The operational definition of brand mixing varied among studies, particularly with respect to the component interfaces used to classify a construct as brand-mixed. Definitions variously considered manufacturer mismatch across the stem, femoral head, and acetabular components or specifically between the femoral and acetabular components.

Baseline demographic, clinical, and implant characteristics are summarized in Table 1a–c. Five studies used national arthroplasty registries, and one study used an institutional registry. The included studies were based on distinct registry or institutional data sources from different countries, and no overlap of study populations was identified.

Reporting of demographic and clinical baseline characteristics varied across studies (Table 1b), limiting direct comparison of several variables between cohorts. Age was reported using different formats, while variables such as ASA score, BMI, primary diagnosis, and previous surgery were not consistently available across studies. Follow-up duration and its reporting varied considerably, with maximum reported follow-up extending to 17 years.

Reporting of implant-related characteristics also varied across studies (Table 1c). Cementation method and bearing surface were the most consistently reported variables, although their distributions differed between brand-mixed and brand-unmixed cohorts and across studies. Differences in other implant characteristics, including femoral head size and component configurations, were also reported in individual studies. Approaches to controlling for measured confounding differed across studies and included multivariable regression, propensity score matching, and stratified analyses, while some outcome analyses did not incorporate multivariable adjustment. Details of study-specific adjustment strategies and covariates are provided in Supplementary Table S6.

**Table 1:** (a) Baseline study characteristics. (b) Clinical baseline characteristics. (c) Implant baseline characteristics (percentages only). (Tucker et al. [13], Peters et al. [12], Taylor et al. [14], Trebše et al. [22], Al-Jabri et al. [15], Kirchner et al. [16]).

**(a)**												
	**Tucker et al.**		**Peters et al.**		**Taylor et al.**		**Trebše et al.**		Al-Jabri et al. *		**Kirchner et al.**	
Year	2015		2016		2017		2020		2026		2026	
Country	UK		Netherlands		New Zealand		Slovenia		Canada		USA	
Study design	Retrospective cohort		Retrospective cohort		Retrospective cohort		Retrospective cohort		Retrospective cohort		Retrospective cohort	
Study period	2003–2013		2007–2014		1999–2015		2002–2004		2012–2020		2012–2021	
Follow-up (median) in years	0–10 (NR)		0–8 (3)		0–17 (NR)		0–14 (10.7)		0–2 (NR)		≥2 ** (NR)	
Total THAs, n	478,880		161,360		99,732		1927		9064		434,985	
Brand-mixed, n (%)	85,051 (17.8)		18,396 (11.4)		24,537 (24.6)		1067 (55.4)		824 (9.1)		21,378 (4.9)	
Brand-unmixed, n (%)	393,829 (82.2)		142,964 (88.6)		75,195 (75.4)		860 (44.6)		8240 (90.9)		413,607 (95.1)	
**(b)**												
	**Tucker et al.**		**Peters et al.**		**Taylor et al.**		**Trebše et al.**		Al-Jabri et al. **		**Kirchner et al.**	
	**Mixed**	**Unmixed**	**Mixed**	**Unmixed**	**Mixed**	**Unmixed**	**Mixed**	**Unmixed**	**Mixed**	**Unmixed**	**Mixed**	**Unmixed**
Age *	71 y	65.2 y	70.3% <75 y	71.3% <75 y	77.0% <75 y	72.3% <75 y	66.4 y	65.3 y	60.8% <70 y	60.8% <70 y	74	73
Male (%)	39.0	40.0	33.8	32.8	NR	NR	35.0	39.0	39.8	39.8	37	40
ASA I (%)	NR	NR	28.0	24.7	NR	NR	NR	NR	NR	NR	NR	NR
ASA II (%)	NR	NR	56.8	62.9	NR	NR	NR	NR	NR	NR	NR	NR
ASA III–IV (%)	NR	NR	15.2	12.4	NR	NR	NR	NR	NR	NR	NR	NR
BMI	NR	NR	NR	NR	NR	NR	27.7	28.1	NR	NR	28.5	29.2
Initial Diagnosis (%)												
Osteoarthritis	NR	NR	83.2	99.9	NR	NR	74.8	80.6	NR	NR	NR	NR
Non-osteoarthritis	NR	NR	16.8	0.1	NR	NR	25.2	19.4	NR	NR	NR	NR
Previous operation (%)												
No	NR	NR	88.4	93.9	NR	NR	NR	NR	NR	NR	NR	NR
Yes	NR	NR	8.2	4.9	NR	NR	NR	NR	NR	NR	NR	NR
Unknown	NR	NR	3.3	1.2	NR	NR	NR	NR	NR	NR	NR	NR
**(c)**												
	**Tucker et al.**		**Peters et al.**		**Taylor et al.**		**Trebše et al.**		**Al-Jabri et al.**		**Kirchner et al.**	
	**Mixed**	**Unmixed**	**Mixed**	**Unmixed**	**Mixed**	**Unmixed**	**Mixed**	**Unmixed**	**Mixed**	**Unmixed**	**Mixed**	**Unmixed**
Cemented fixation	56.6	41.8	18.0	71.6	8.9	27.3	27.0	12.0	8.4	7.9	11	5
Uncemented fixation	12.4	44.6	40.3	15.7	26.1	42.7	NR	NR	NR	NR	89	95
Hybrid fixation	30.9	13.6	41.7	12.7	65.0	30.1	NR	NR	NR	NR	NR	NR
Bearing type												
C/MoP	93.0	70.5	87.7	81.4	90.7	81.4	80.0	75.1	NR	NR	NR	NR
CoC	4.5	22.0	9.2	8.5	8.0	11.4	18.6	17.4	NR	NR	NR	NR
MoM	2.4	7.5	0.6	4.5	0.9	6.7	0.0	0.0	NR	NR	NR	NR
CoM	NR	NR	NR	NR	0.3	0.5	NR	NR	NR	NR	NR	NR
Other	NR	NR	2.5	5.6	NR	NR	1.5	7.5	NR	NR	NR	NR
Dual mobility	NR	NR	NR	NR	NR	NR	NR	NR	NR	NR	27	7
Head size												
22–28 mm	NR	NR	58.9	37.5	1.8	1.1	NR	NR	NR	NR	3	3
32 mm	NR	NR	29.9	41.9	69.5	65.5	NR	NR	NR	NR	18	25
36 mm	NR	NR	8.8	18.4	21.2	28.3	NR	NR	NR	NR	72	65
>36 mm	NR	NR	2.4	2.2	7.5	5.1	NR	NR	NR	NR	8	7

Note for (a): Abbreviations: THA = total hip arthroplasty. * Propensity score-matched cohort used for outcome analysis. ** Minimum follow-up of 2 years; revision status was captured through December 2023. Note for (b): Abbreviations: ASA = American Society of Anesthesiologists physical status classification system; BMI = body mass index; y = years. * Age reported as mean (years) or proportion below the specified age threshold. ** Al-Jabri et al. data are presented for the propensity score-matched cohort. Note for (c): Abbreviations: C/MoP = ceramic- or metal-on-polyethylene; CoC = ceramic-on-ceramic; MoM = metal-on-metal; CoM = ceramic-on-metal. General note: Abbreviations: NR = not reported. Throughout Table 1a–c, “mixed” and “unmixed” refer to brand-mixed and brand-unmixed THA, respectively.

### 3.3. Quality and Certainty of Evidence Assessment

MINORS [20] scores for individual studies are presented in Table S2. Scores ranged from 18 to 19 out of 24. All studies were retrospective cohort studies, and revision endpoints were collected independently of study investigators, consistent with MINORS criteria for unbiased endpoint assessment.

According to the GRADE [21] framework, the certainty of evidence was rated low for the time-to-event analysis and very low for both the crude revision-event analysis and the post hoc exploratory contemporary-construct sensitivity analysis. The time-to-event analysis showed no statistical heterogeneity (I^2^ = 0%) and was not downgraded beyond the initial low certainty assigned to observational evidence. The crude revision-event analysis was downgraded for potential residual confounding and substantial inconsistency (I^2^ = 93%). The contemporary-construct sensitivity analysis was similarly limited by potential residual confounding and substantial inconsistency (I^2^ = 95%) and was based on only two studies. Detailed certainty assessments are provided in Table S3 (Summary of Findings).

### 3.4. Primary Outcome

#### 3.4.1. Time-to-Event Analysis

**Figure 3:**
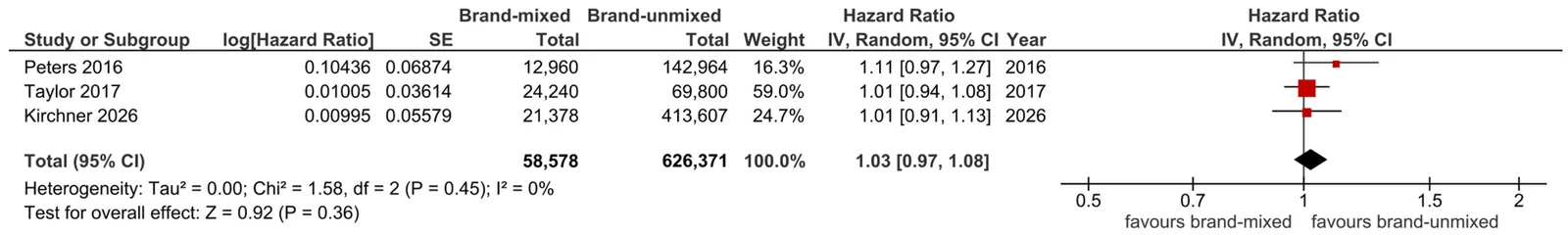
Forest plot of the time-to-event analysis comparing all-cause revision between brand-mixed and brand-unmixed primary total hip arthroplasty (Peters et al. [12], Taylor et al. [14], Kirchner et al. [16]). Squares represent individual study estimates (size proportional to study weight), horizontal lines indicate 95% confidence intervals, and diamonds represent pooled estimates with their corresponding 95% confidence intervals. Abbreviations: HR = hazard ratio; CI = confidence interval.

Three studies reported suitable hazard ratios for all-cause revision and were included in the time-to-event meta-analysis [12,14,16]. The principal analysis included the adjusted mixed head–cup estimate from Peters et al. [12], the estimate from the cohort excluding metal-on-metal and ceramic-on-metal bearings from Taylor et al. [14], and the adjusted overall estimate from Kirchner et al. [16]. The pooled analysis did not identify a statistically significant association between brand mixing and all-cause revision (HR 1.03, 95% CI 0.97–1.08; *p* = 0.36), with no observed statistical heterogeneity (I^2^ = 0%; Figure 3). Sensitivity analyses using alternative eligible HR estimates yielded pooled HRs ranging from 0.99 to 1.01, with all 95% confidence intervals including the null (Table S4).

#### 3.4.2. Crude Revision-Event Analysis

Five studies provided extractable revision-event counts and were included in the complementary crude revision-event meta-analysis.

**Figure 4:**
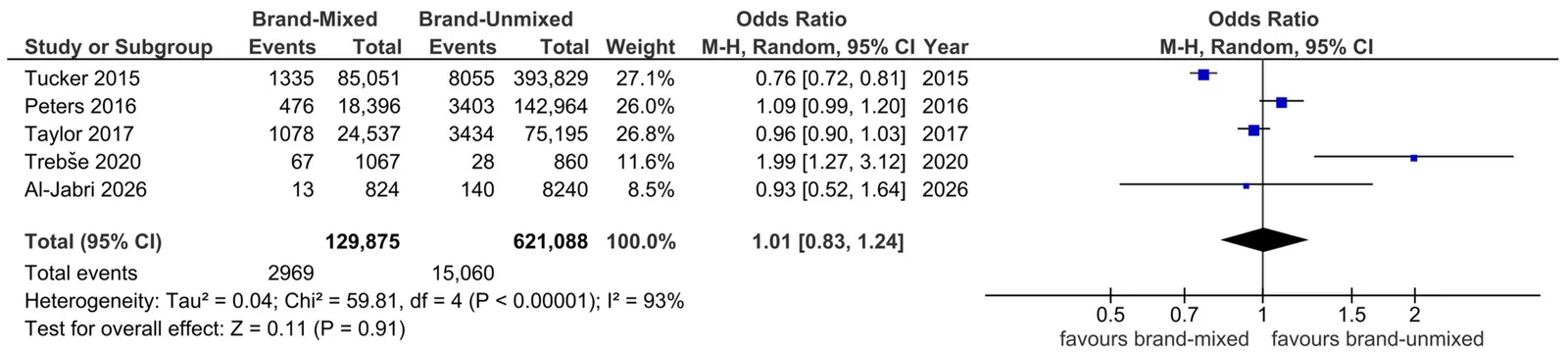
Forest plot of the crude revision-event analysis comparing all-cause revision between brand-mixed and brand-unmixed primary total hip arthroplasty (Tucker et al. [13], Peters et al. [12], Taylor et al. [14], Trebše et al. [22], Al-Jabri et al. [15]). Squares represent individual study estimates (size proportional to study weight), horizontal lines indicate 95% confidence intervals, and diamonds represent pooled estimates with their corresponding 95% confidence intervals. Abbreviations: OR = odds ratio; CI = confidence interval.

Across the five studies, crude revision proportions were 2.3% (2969/129,875) for brand-mixed and 2.4% (15,060/621,088) for brand-unmixed THA. The pooled analysis did not identify a statistically significant difference in all-cause revision between groups (OR 1.01, 95% CI 0.83–1.24; *p* = 0.91; Figure 4). Statistical heterogeneity was substantial (I^2^ = 93%), with individual study estimates varying in both magnitude and direction. A leave-one-out sensitivity analysis showed that exclusion of individual studies did not materially alter the pooled effect estimate, with ORs ranging from 0.93 to 1.09 and all 95% confidence intervals including the null. Substantial heterogeneity persisted across these analyses (I^2^ = 77–95%), indicating that the observed heterogeneity was not attributable to a single study (Table S5). Detailed revision-event data by study are provided in Table 2.

#### 3.4.3. Exploratory Contemporary-Construct Sensitivity Analysis

**Figure 5:**
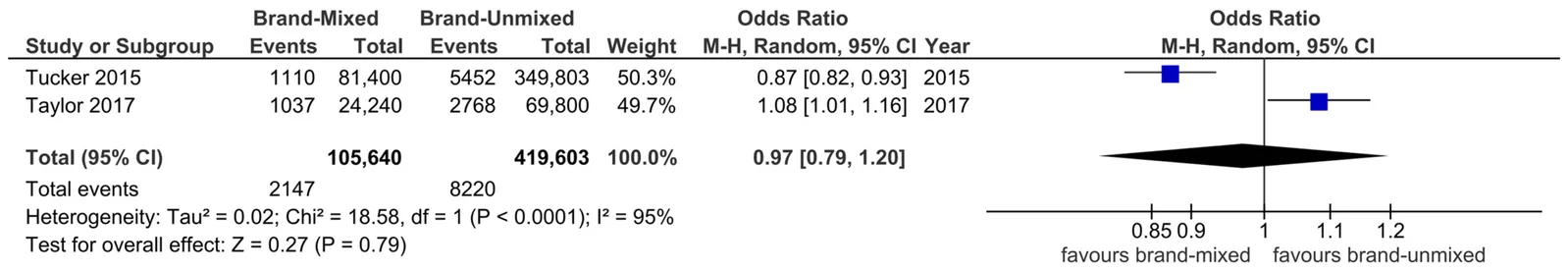
Forest plot of the post hoc exploratory contemporary-construct sensitivity analysis comparing all-cause revision between brand-mixed and brand-unmixed primary total hip arthroplasty (Tucker et al. [13], Taylor et al. [14]). Squares represent individual study estimates (size proportional to study weight), horizontal lines indicate 95% confidence intervals, and diamonds represent pooled estimates with their corresponding 95% confidence intervals. Abbreviations: OR = odds ratio; CI = confidence interval.

A post hoc exploratory sensitivity analysis was performed using contemporary constructs, excluding metal-on-metal and ceramic-on-metal bearings and brand-mixed stem–head combinations. Two studies provided sufficient data for this analysis. Crude revision proportions were 2.03% (2147/105,640) for brand-mixed and 1.96% (8220/419,603) for brand-unmixed THA. The pooled analysis did not identify a statistically significant difference in all-cause revision between groups (OR 0.97, 95% CI 0.79–1.20; *p* = 0.79; Figure 5). Statistical heterogeneity remained substantial (I^2^ = 95%), with the two study estimates differing in direction.

### 3.5. Secondary Outcomes

#### 3.5.1. Reasons for Revision

Four studies reported indications or cause-specific outcomes for revision (Table 2). Reported causes included dislocation or instability, infection, aseptic loosening, periprosthetic fracture, wear, pain, and leg-length discrepancy. Peters et al. [12] reported dislocation as the most frequent indication for revision, occurring in 0.8% of brand-mixed and 0.7% of brand-unmixed THAs. Al-Jabri et al. [15] found no significant difference in revision indications between groups, while Kirchner et al. [16] found no significant association between component mixing and cause-specific revision for aseptic loosening, infection, instability, or periprosthetic fracture. Because reporting of revision indications varied across studies and cause-specific outcomes were not consistently available, these outcomes were not quantitatively pooled. No clear pattern of differences in revision indications between brand-mixed and brand-unmixed THA was apparent from the available descriptive data.

**Table 2:** All-cause revision and indications for revision surgery (%).

	Tucker et al.		Peters et al.		Taylor et al.		Trebše et al.		Al-Jabri et al. †	
Indication	Mixed	Unmixed	Mixed	Unmixed	Mixed	Unmixed	Mixed	Unmixed	Mixed	Unmixed
All-cause revision	1.6	2.0	2.6	2.4	4.4	4.6	6.3	3.3	1.6	1.7
Septic/infection	NR	NR	0.3	0.3	NR	NR	1.1	0.1	<0.7	0.6
Aseptic revision (overall)	NR	NR	NR	NR	NR	NR	5.2	3.1	NR	NR
Dislocation	NR	NR	0.8	0.7	NR	NR	0.2 *	0.6 *	<0.7	0.6
Femoral loosening	NR	NR	0.5	0.5	NR	NR	NR	NR	NR	NR
Acetabular loosening	NR	NR	0.4	0.3	NR	NR	NR	NR	NR	NR
Symptomatic MoM bearing	NR	NR	0.04 **	0.2 **	NR	NR	NR	NR	NR	NR
Periprosthetic fracture	NR	NR	0.3	0.3	NR	NR	NR	NR	<0.7	0.3
Cup/liner wear	NR	NR	0.1	0.1	NR	NR	NR	NR	NR	NR
Periarticular ossification	NR	NR	0.1	0.1	NR	NR	NR	NR	NR	NR
Girdlestone	NR	NR	0.1	0.1	NR	NR	NR	NR	NR	NR
Other	NR	NR	0.4	0.4	NR	NR	NR	NR	NR	NR
Ceramic breakage	NR	NR	NR	NR	NR	NR	0.9 *	0.3 *	NR	NR

Abbreviations: MoM = metal-on-metal; NR = not reported. Values represent crude (unadjusted) percentages of the respective THA cohorts unless otherwise indicated. For brevity, “mixed” and “unmixed” refer to brand-mixed and brand-unmixed THA, respectively. * Dislocation and ceramic breakage were included within aseptic revisions in Trebše et al. ** MoM bearings were overrepresented in the brand-unmixed group in Peters et al. † Al-Jabri et al. data are presented for the propensity score-matched cohort; cell counts <6 were suppressed by the authors and are reported as the corresponding percentage limits. Kirchner et al. [16] reported cause-specific revision outcomes as hazard ratios rather than absolute percentages, and therefore this study is not presented in this table. Values < 0.1% are shown with two decimals. The sum of reported revision indications in Peters et al. differs from the reported total number of revisions; the reason for this discrepancy was not specified. (Tucker et al. [13], Peters et al. [12], Taylor et al. [14], Trebše et al. [22], Al-Jabri et al. [15]).

#### 3.5.2. Other Reported Outcomes

Other clinical outcomes were reported inconsistently across studies and were not suitable for quantitative synthesis. Taylor et al. [14] reported mean Oxford Hip Scores at six months of 41.1 in the brand-mixed and 40.3 in the brand-unmixed group. Trebše et al. [22] reported a longer mean operative time in the brand-mixed group (63.1 vs 53.1 min), while Al-Jabri et al. [15] reported a shorter mean length of hospital stay in the propensity score–matched brand-mixed cohort (2.84 vs 3.23 days, *p* < 0.0001). These findings are summarized in Table 3.

## 4. Discussion

In this meta-analysis of six retrospective cohort studies, the available evidence did not identify a statistically significant association between brand mixing and all-cause revision following primary THA. The time-to-event analysis, which was given greater interpretative emphasis because it accounts for differences in follow-up and censoring, yielded a pooled HR of 1.03 (95% CI 0.97–1.08), with no observed statistical heterogeneity (I^2^ = 0%). Sensitivity analyses using alternative eligible HR estimates did not materially alter this finding. The complementary crude revision-event analysis likewise did not identify a statistically significant difference between brand-mixed and brand-unmixed THA (OR 1.01, 95% CI 0.83–1.24), but substantial heterogeneity was present (I^2^ = 93%). Leave-one-out analyses produced pooled ORs ranging from 0.93 to 1.09, while heterogeneity remained substantial (I^2^ = 77–95%), indicating that the heterogeneity was not attributable to a single study. Overall, neither analytical approach identified a statistically significant association between brand mixing and all-cause revision; however, these findings do not establish equivalence between brand-mixed and brand-unmixed constructs.

The difference in heterogeneity between the time-to-event and crude revision-event analyses likely reflects, at least in part, substantial methodological and clinical differences among the included studies. Follow-up duration, study period, patient characteristics, fixation, bearing surfaces, femoral head sizes, and the component interfaces used to define brand mixing varied across studies. In addition, approaches to controlling for confounding differed considerably. Peters et al. [12] and Kirchner et al. [16] provided multivariable-adjusted time-to-event estimates, whereas other studies used propensity score matching, stratification, or predominantly unadjusted comparisons. The pooled crude ORs were based on aggregate event counts and therefore did not preserve study-level multivariable adjustment, although the Al-Jabri et al. [15] event counts were derived from a propensity score–matched cohort. Because component selection was not randomized, the decision to use a brand-mixed construct may have been associated with patient-, implant-, surgeon-, or institution-related factors that also influence revision risk. Residual confounding and confounding by indication or selection therefore cannot be excluded.

The specific component interface being mixed may also be clinically relevant. Previous biomechanical studies have demonstrated differences in taper geometry and connection strength among cross-manufacturer stem–head combinations, raising concerns regarding mechanical stability and potential fretting corrosion, which may contribute to metal debris generation and adverse local tissue reactions [10,11]. Similarly, the choice of bearing surface, particularly the use of metal-on-metal (MoM) articulations, may independently influence revision risk [23]. Metallic wear debris and associated cobalt and chromium ion release may contribute to local inflammatory responses, metallosis, osteolysis, and pseudotumor formation, while elevated circulating metal ion concentrations raise concerns regarding potential systemic effects [24]. Long-term registry data have demonstrated an increased risk of metallosis-related failure associated with large-diameter MoM bearings [25]. In the included studies, the distribution of such constructs differed between brand-mixed and brand-unmixed cohorts. For example, Trebše et al. [22] reported that most brand-mixed constructs involved stem–head mixing, whereas metal-on-metal bearings were disproportionately represented in some brand-unmixed cohorts. We therefore performed a post hoc exploratory contemporary-construct sensitivity analysis excluding metal-on-metal and ceramic-on-metal bearings and brand-mixed stem–head combinations. This analysis did not identify a statistically significant difference in revision (OR 0.97, 95% CI 0.79–1.20), but it included only two studies and retained substantial heterogeneity (I^2^ = 95%). It should therefore be regarded as exploratory and does not establish the safety of particular component combinations.

The present findings extend the previously available literature. During preparation of this study, Leitner et al. [26] published a systematic review addressing brand mixing in primary THA. That review provided a qualitative synthesis but did not quantitatively pool revision outcomes. The current review incorporates additional recently published cohorts and provides quantitative synthesis using both time-to-event estimates and revision-event data.

Indications for revision were reported inconsistently, and differences in outcome definitions and reporting prevented quantitative synthesis of individual revision causes. Similarly, functional and other clinical outcomes were sparsely reported: only one included study provided a functional outcome measure, while operative duration and length of hospital stay were each reported by individual studies. The available evidence therefore primarily informs all-cause revision risk and does not permit robust conclusions regarding functional outcomes, specific causes of revision, or other perioperative outcomes.

Several limitations should be considered. First, all six included studies were retrospective observational cohorts. Although some studies used multivariable adjustment or propensity score matching, adjustment strategies and included covariates differed, and residual confounding and confounding by indication or selection cannot be excluded. Furthermore, although MINORS was used as prespecified to assess methodological quality, it provides a broad study-level assessment and has limited granularity for evaluating specific bias domains, particularly residual confounding. Second, follow-up duration varied substantially. Crude revision-event proportions do not account for time at risk or censoring, which was the rationale for giving greater interpretative emphasis to the time-to-event analysis. However, only three studies contributed suitable HRs, and these estimates were not uniformly multivariable-adjusted. The time-to-event analysis and the hierarchy used to select among multiple eligible HR estimates were also introduced post hoc rather than prespecified in the registered analysis plan, although sensitivity analyses using alternative eligible estimates yielded similar pooled effects. Third, substantial heterogeneity in the crude revision-event analysis remained after sequential exclusion of individual studies, indicating broader differences in populations, implant constructs, study periods, follow-up, and analytical methods rather than the influence of a single study. The exploratory contemporary-construct analysis was additionally limited by inclusion of only two studies and substantial heterogeneity. Fourth, despite the large aggregate sample of more than 1.18 million THAs, the evidence was derived from only six studies, five based on national or population-based registry data and one on an institutional registry. No overlap of study populations was identified because the studies used distinct registry data sources from different countries. The large pooled sample therefore provides substantial statistical precision but should not be interpreted as increasing the certainty of the observational evidence. Fifth, aggregate data did not permit robust analyses of individual manufacturer combinations, specific component interfaces, bearing materials, fixation methods, or other implant characteristics. Finally, the search was restricted to English-language published studies in PubMed and Embase, supplemented by reference-list screening; grey literature, conference abstracts, and trial registries were not systematically searched. Relevant unpublished or non-English studies may therefore have been missed, and the small number of included studies precluded meaningful formal assessment of publication bias.

These limitations are reflected in the GRADE assessment. Certainty of evidence was rated low for the time-to-event analysis and very low for both the crude revision-event analysis and the post hoc exploratory contemporary-construct sensitivity analysis. The latter analyses were particularly limited by substantial inconsistency, in addition to the potential for residual confounding inherent in the observational evidence. Accordingly, the absence of statistically significant associations should not be interpreted as evidence of equivalence, universal component compatibility, or the safety of specific brand-mixed combinations.

Regulatory and medico-legal considerations remain relevant independent of the observed revision outcomes. Cross-manufacturer component combinations may fall outside manufacturer-specified compatibility and may not have undergone standardized testing as a construct. Consequently, surgeons should consider applicable regulatory requirements, manufacturer recommendations, component compatibility, and documentation when selecting such combinations. Peters et al. [27] discussed the European legal context; however, the absence of an observed increase in revision risk at the population level does not itself resolve regulatory or liability considerations. More standardized compatibility testing and clearer guidance could facilitate evidence-based decisions regarding specific cross-manufacturer combinations [28].

Future research should prioritize prospective or high-quality registry-based time-to-event analyses with standardized adjustment for relevant patient-, implant-, surgeon-, and institution-level confounders. Registries should capture specific component interfaces, manufacturer combinations, bearing materials, fixation methods, head sizes, and other relevant implant characteristics. Such data would allow evaluation of individual component combinations rather than treating brand mixing as a single exposure and may help identify combinations associated with higher or lower revision risk.

## 5. Conclusions

The available observational evidence did not identify a statistically significant association between brand mixing and all-cause revision following primary THA. The time-to-event analysis showed no observed statistical heterogeneity, whereas substantial heterogeneity was present in the crude revision-event and post hoc exploratory contemporary-construct analyses. The certainty of evidence was low to very low, and these findings do not establish equivalence, universal component compatibility, or the safety of specific brand-mixed combinations. Further long-term studies with detailed reporting of individual component combinations and standardized time-to-event analyses are needed to better characterize the revision risk associated with specific brand-mixed constructs.

## Figures and Tables

**Table 3:** Other reported outcomes.

Outcome	Study	Mixed	Unmixed
Operative time (min), mean	Trebše et al.	63.1	53.1
Oxford Hip Score at 6 months, mean	Taylor et al.	41.1	40.3
Length of hospital stay (days), mean ± SD	Al-Jabri et al.	2.84 ± 2.25	3.23 ± 2.29

Abbreviations: SD = standard deviation. For brevity, “mixed” and “unmixed” refer to brand-mixed and brand-unmixed THA, respectively. (Taylor et al. [14], Trebše et al. [22], Al-Jabri et al. [15]).

## Data Availability

All data analyzed in this systematic review and meta-analysis were derived from previously published studies. Data used for the analyses are presented in the manuscript and Supplementary Materials. Additional extracted data and data collection materials are available from the corresponding author upon reasonable request.

## Supplementary Materials

The following supporting information can be downloaded at: https://www.mdpi.com/article/10.3390/jcm15207904/s1, PRISMA 2020 CHECKLIST, Table S1: Complete electronic search strategy for PubMed/MEDLINE and Embase; Table S2: Methodological quality assessment using the MINORS criteria; Table S3: Summary of findings (GRADE); Table S4: Sensitivity analyses of alternative effect-estimate selection in the time-to-event meta-analysis; Table S5: Leave-one-out sensitivity analysis of the crude revision-event meta-analysis; Table S6: Confounding-control and adjustment strategies in the included studies.

## Abbreviations

The following abbreviations are used in this manuscript:

ASA	American Society of Anesthesiologists physical status classification system
BMI	body mass index
CI	confidence Interval
CoC	ceramic-on-ceramic
CoM	ceramic-on-metal
GRADE	Grading of Recommendations Assessment, Development and Evaluation
HR	hazard ratio
MINORS	Methodological Index for Non-Randomized Studies
MoM	metal-on-metal
MOOSE	Meta-analysis of Observational Studies in Epidemiology
OR	odds ratio
PRISMA	Preferred Reporting Items for Systematic Reviews and Meta-Analyses
PROSPERO	International Prospective Register of Systematic Reviews
SD	standard deviation
THA	total hip arthroplasty
