# Supplementary material for: Meta-Analysis of Brand-Mixed Versus Brand-Unmixed Primary Total Hip Arthroplasty
Source: J Clin Med. 2026 Oct 13;15(20):7904. doi: 10.3390/jcm15207904 (PMC13705420; doi:10.3390/jcm15207904)
Supplement: Supplementary file 1 [file jcm-15-07904-s001.zip › jcm-4529224-supplementary.pdf]

Journal of Clinical Medicine

Systematic review

Meta-Analysis of Brand-Mixed Versus Brand-Unmixed Primary Total Hip Arthroplasty

Daniel Elias [1,2]*, Bryan J. M. van de Wall [1,2], Jörg Bucher [1], Björn-Christian Link [1,2] and Pascal C. Haefeli [1,2]

[1] Department of Orthopedic and Trauma Surgery, Lucerne Cantonal Hospital, 6004 Lucerne, Switzerland bryan.vandewall@luks.ch (B.J.M.v.d.W.); joerg.bucher@luks.ch (J.B.); bjoern-christian.link@luks.ch (B.-C.L.); pascal.haefeli@luks.ch (P.C.H.)
[2] Department of Health Science and Medicine, University of Lucerne, 6005 Lucerne, Switzerland
* Correspondence: daniel.elias@stud.unilu.ch; Tel.: 0041766050156

Supplementary Material

Database	Search terms	Filters Applied	Date of Search	Results
PubMed/MEDLINE	("total hip arthroplasty" OR "total hip replacement" OR "hip replacement surgery") AND ("different manufacturers" OR "matched components" OR "unmatched components" OR "mixing components" OR mismatch OR mix OR non-compatible OR "off-label" OR "mix and match")	English language; Humans	10 September 2026	278
Embase	("total hip arthroplasty") AND ("different manufacturers" OR "matched components" OR "mixing components" OR non-compatible OR "off-label" OR "mix and match")	Review and research articles only; English language	11 September 2026	70

Table S1. Complete electronic search strategy for PubMed/MEDLINE and Embase.

Notes: Search strategies were applied to titles, abstracts, and keywords. No date restrictions were applied. Searches were performed separately in PubMed/MEDLINE and Embase.
The initial searches were conducted in PubMed on 4 September 2024 and in Embase on 20 September 2024. The searches were updated using the same strategy on 10 September 2026 and 11 September 2026, respectively; the table reports the updated search results.

Table S2. Methodological quality assessment using the MINORS criteria.

	Tucker et al.	Peters et al.	Taylor et al.	Trebše et al.	Al Jabri et al.	Kirchner et al.
Clearly stated aim	2	2	2	2	2	2

 https://doi.org/10.3390/jcm15207904

Inclusion of consecutive patients	2	2	2	2	2	2
Prospective data collection	0	0	0	0	0	0
Appropriate endpoints	2	2	2	2	2	2
Unbiased assessment of endpoints	2	2	2	2	2	2
Appropriate follow-up (>1 year)	2	2	2	2	2	2
Loss to follow-up <5%	1	1	2	2	1	2
Prospective calculation of study size	0	0	0	0	0	0
Adequate control group	2	2	2	2	2	2
Contemporary groups	2	2	2	2	2	2
Baseline equivalence of groups	1	1	1	1	2	1
Adequate statistical analyses	2	2	2	1	2	2
Total MINORS score	18	18	19	18	19	19

Abbreviations: MINORS, Methodological Index for Non-Randomized Studies.
Scores per item: 0 = not reported; 1 = reported but inadequate; 2 = reported and adequate. Maximum possible score for comparative studies = 24.

Table S3. Summary of Findings (GRADE).

	**All-cause revision - time-to-event analysis**	**All-cause revision – crude revision-event analysis**	**All-cause revision - exploratory contemporary-construct sensitivity analysis**
Hips (studies)	684,949 hips (3 studies)	750,963 hips (5 studies)	525,243 hips (2 studies)

https://doi.org/10.3390/jcm15207904

Studies	Peters et al.; Taylor et al.; Kirchner et al.	Tucker et al.; Peters et al.; Taylor et al.; Trebše et al.; Al-Jabri et al.	Tucker et al.; Taylor et al.
Study designs	Retrospective cohort studies	Retrospective cohort studies	Retrospective cohort studies
Relative effect (95% CI)	HR 1.03 (0.97-1.08)	OR 1.01 (0.83-1.24)	OR 0.97 (0.79-1.20)
Heterogeneity	I^2 = 0%	I^2 = 93%	I^2 = 95%
Certainty (GRADE)	⊕⊕◯◯ Low	⊕◯◯◯ Very low	⊕◯◯◯ Very low
Certainty assessment	Low certainty. No further downgrading beyond the initial low certainty assigned to observational evidence; no statistical heterogeneity was observed (I^2 = 0%).	Very low certainty due to the observational study designs with potential residual confounding and serious inconsistency (I^2 = 93%).	Very low certainty due to the observational study designs with potential residual confounding and serious inconsistency (I^2 = 95%). The analysis was post hoc and based on only two studies.

Abbreviations: CI = confidence interval; OR = odds ratio; HR = hazard ratio; I^2 = percentage of variability due to heterogeneity rather than chance; GRADE = Grading of Recommendations Assessment, Development and Evaluation.

Note: Certainty of evidence was assessed according to the GRADE framework. Observational studies started at low certainty and were further downgraded for serious risk of bias, inconsistency, indirectness, imprecision, or publication bias, where applicable.

Table S4. Sensitivity analyses of alternative effect-estimate selection in the time-to-event meta-analysis

Analysis	Effect estimate selected	Pooled HR	95% CI	I^2
Principal analysis	Peters: adjusted mixed head-cup; Taylor: MoM/CoM-excluded cohort	1.03	0.97-1.08	0%
Sensitivity analysis 1	Peters: adjusted mixed stem-head	0.99	0.90-1.07	39%
Sensitivity analysis 2	Peters: adjusted mixed stem-head-cup	1.01	0.95-1.07	0%
Sensitivity analysis 3	Taylor: entire study cohort	1.00	0.91-1.10	63%

Abbreviations: CI = confidence interval; CoM = ceramic-on-metal; HR = hazard ratio; MoM = metal-on-metal; THA = total hip arthroplasty.

All other study-level estimates were retained as in the principal analysis. HRs were harmonized to represent brand-mixed versus brand-unmixed THA. The time-to-event analysis and effect-estimate selection hierarchy were not prespecified in the registered analysis plan and were performed post hoc.

Table S5. Leave-one-out sensitivity analysis of the crude revision-event meta-analysis.

Study excluded	OR	95% CI	I^2
Tucker et al.	1.09	0.92-1.30	77%
Peters et al.	0.98	0.78-1.24	92%
Taylor et al.	1.06	0.78-1.45	94%
Trebše et al.	0.93	0.76-1.12	94%
Al-Jabri et al.	1.02	0.83-1.26	95%

Abbreviations: CI = confidence interval; OR = odds ratio.
Each analysis was performed after sequential exclusion of one study from the crude revision-event meta-analysis using the same random-effects Mantel–Haenszel model as in the full five-study analysis.

Table S6. Confounding-control and adjustment strategies in the included studies

Study	Adjustment approach	Variables included	Estimate/data used in the meta-analysis
Tucker et al.	Age- and sex-stratified survival analyses	Age, sex	Crude event counts
Peters et al.	Multivariable Cox regression	Age, sex, ASA, diagnosis, previous operation, fixation, head diameter, articulation, surgical period	Adjusted HR; crude event counts
Taylor et al.	Cox regression; additional multivariable analysis	Age, bearing surface, fixation, head size	Reported HR*; crude event counts
Trebše et al.	No multivariable adjustment; Kaplan–Meier/log-rank and bivariate analyses	None (no multivariable adjustment reported)	Crude event counts
Al-Jabri et al.	1:10 propensity score matching	Age, sex, Charlson Comorbidity Index, residence, marginalization indicators, discharge destination, bone cement, hospital type	Crude event counts from propensity score–matched cohort
Kirchner et al.	Multivariable cause-specific Cox regression	Age, sex, BMI, Charlson Comorbidity Index	Adjusted HR

Abbreviations: ASA = American Society of Anesthesiologists; BMI = body mass index; HR = hazard ratio.

https://doi.org/10.3390/jcm15207904

* The HR used from Taylor et al. was not clearly reported as a multivariable-adjusted estimate; the study additionally evaluated age, bearing surface, fixation, and head size in a multivariable model.

https://doi.org/10.3390/jcm15207904
